# Horizontal Gene Transfer and Its Part in the Reorganisation of Genetics during the LUCA Epoch 

**DOI:** 10.3390/life3040518

**Published:** 2013-10-28

**Authors:** Sohan Jheeta

**Affiliations:** Network of Researchers HGT & LUCA, 1 Scott Hall Crescent, Leeds, LS7 3RB, UK; E-Mail: sohan7@ntlworld.com; Tel.: +44-113-262-8767

**Keywords:** Horizontal gene transfer, lateral gene transfer, phylogenetic reconstruction transformation, transduction, conjugation, gene transfer agent (GTA), membrane vesicle transfer (MVT), phylogenetic tree of life, origin of life, last universal common ancestor (LUCA)

## Abstract

Currently there are five known mechanisms of horizontal gene transfer (HGT): transduction, conjugation, transformation, gene transfer agents and membrane vesicle transfer. The question here is: what part did HGT play in the reorganisation of genetics during the last universal common ancestor (LUCA) epoch? LUCA is a construct to explain the origin of the three domains of life; namely Archaea, Bacteria and Eukarya. This editorial offers a general introduction to the relevance and ultimate significance of HGT in relation to the LUCA.

## 1. Introduction

Horizontal gene transfer (HGT) is the transfer/exchange of genetic material between extant donor and recipient cells or the active take-up of strands of “naked” nucleic acids from the environment by competent cells, which are then themselves transformed. This is opposed to vertical gene transfer (VGT), the transfer of genes from parent to progeny. HGT is a ubiquitous mechanism, particularly prevalent in microbes; it occurs between the cells of the same genus as well between different genera and even different domains of life. HGT is an important process in modern biological systems—for example it has been shown that the modification of photosynthetic apparatus in cyanobacteria is widespread in marine phytoplankton as was demonstrated by the recent identification of photosystem I (PSI) and photosystem II (PS II) genes in viruses (cyanophages) of marine cyanobacteria. In this case, cyanophages seemingly help to optimise photosynthesis by providing additional photosystem genes with different properties. Having defined the parameters of HGT, the question which we aim to address in this special issue is: “what part did HGT play during the reorganisation of genomes during the era of the last universal common ancestor (LUCA) which led to formation of the three main domains of life?”

It is the focus of this special issue to consider the involvement of HGT in relation to the LUCA, beginning with the formation of nucleic acids and the subsequent emergence of the LUCA, prior to the three domains becoming distinguished as separate branches in the development of life. There is good evidence for ancient HGT events, indicating that the evolutionary history of genes within genomes is best understood in terms of networks. Thus, we invite articles that consider the extent to which HGT contributed to the evolution of LUCA and the very early evolution of life on Earth. 

## 2. The HGT Phenomenon

The importance of HGT cannot be underestimated as it is probable that life on Earth as we know it today would not be likely to exist without it. One example being transduction, as viruses represent the largest pool of genetic diversity, with an estimated 10^30^ viruses in the oceans alone, which far exceeds the total number of different types of living organisms on Earth (10^8^); although this is perhaps only a small proportion of the actual total, it is unlikely to be anywhere near as high as 10^30^. There are reported to be a staggering 10^29^ viral infections per day and even if we speculate that only one millionth of 1% of these are involved in transduction, this is still quite a feat in itself.

To explore the phenomenon of HGT we can begin by stating that genes, being the controller of metabolisms (at least in terms of modern biology), are preserved in all life forms. These manifestations of the “controller” molecules can be observed in the unchanging biochemistry of life since, even if the controller itself mutates, overall the molecules of life and the pathways in which they are involved, do not.

What were the initial controller molecules? There are three possibilities; either DNA or RNA, or perhaps an unknown precursor molecule—such as threose nucleic acid (TNA). We don’t know much about TNA or an analogous molecule because, if it was part of initial chemistry, it was not preserved and is lost for all eternity; the best we could do is to draw-up a list of candidates with the relevant properties which could have set in motion the route to the formation of first genes. DNA is also an unlikely precursor because today it is made from RNA by replacing ribose with deoxyribose sugar and uracil nucleobase with thymine; to synthesise DNA *de novo* is exceptionally difficult because there are no known simple mechanisms by which it could be made. Some scientists believe that DNA arose at or during the first branch of the phylogenetic tree of life and that it probably arose twice independently. That is, DNA would have emerged after the appearance of RNA, and in this view RNA must have been dominant during the LUCA era. Although DNA is much more stable compared to RNA, the latter has two essential requirements which “trump” DNA, in that it can act both as a repository for genetic information and as a catalyst (ribozyme). Additional circumstantial evidence in favour of RNA being the initial controller can be gleaned from the tree as well as from RNA viruses (e.g., Retroviruses) present at the time of LUCA. 

In fact, on the basis of biochemical/genetic analysis it can be surmised that virus particles in existence at the time of LUCA contained RNA, with both DNA and DNA replication machineries emerging later. This conclusion can be reached because some viruses share homologous capsid proteins and/or ATPase proteins for packaging, suggesting that they evolved from a virus that existed at the time of or just prior to LUCA. With this in mind, it is believed that RNAs were involved in HGT and thus hold clues to the understanding of the reorganisation of genetics that led to the emergence of the three domains of life, namely Archaea, Bacteria and Eukarya.

## 3. Phylogenetic Tree of Life

The idea of phylogenetic tree of life was first suggested by Charles Darwin under a less fanciful title of “common ancestry” meaning that every living species on Earth arose from a single common organism, which is now referred to as LUCA. But the hard empirical evidence for the phylogenetic tree began with the pioneering work of Woese and Fox [1], who compared the small subunits of ribosomal RNA (16/18S rRNA) from different species and found commonality of a single origin, as was predicted by Charles Darwin. Today, more evidence of divergence from a common “root” has been confirmed by genes such as those encoding for polymerases, ATPase subunits, elongation factors and ribosomal proteins. However, even with such overwhelming evidence in favour of LUCA giving rise to the three domains, it is still a matter of conjecture and debate: Cavalier-Smith [2] argues that “…prokaryotes constitute a single kingdom, Bacteria, here divided into two new subkingdoms: Negibacteria, with a cell envelope of two distinct genetic membranes, and Unibacteria, comprising the new phyla Archaebacteria and Posibacteria…”; the validity of the tree could be further called into question by asking, did HGT lead to the acquisition of physiological properties or metabolic traits that are not concordant with their 16S RNA?; there is also a complication arising from the origin of Eukarya and Archaea, as they have a common history, *i.e.*, did Bacteria lead to their origin, or did proto-eukaryotes give rise to Bacterial and Archaeal domains? Finally, Prof. Micahel Syvanen (see summary report in this issue) demonstrated that, by reworking the phylogenetic tree, the LUCA may not be an appropriate device when describing the origin of its three branches.

The existence of a common genetic code is one of the most compelling evidence in support of the phylogenetic tree and so in light of this there appears to be a common ancestry, leading to the prediction of at least three domains of life; these being possibly derived from viruses and LUCAs which intermingled freely and exchanged MGEs frequently. 

## 4. What is LUCA?

The LUCA is a moot point in that it is a theoretical construct designed to explain the origin of especially the Bacteria and Archaea domains, collectively called prokaryotes. It is believed that LUCA existed at the time that these two domains became separate entities in their own right. From this it can be surmised that LUCA may have been a complex and almost fully formed living entity which probably even had DNA as a repository for information, as has been shown by various comparative genomic studies. More to the point, Prof. John Allen (Queen Mary, University of London—see summary report) proposed: what does the word “last” in “last universal common ancestor” signify? There are two lines of thought on this question, *i.e.*, whether LUCA means the ancestor of all things alive on Earth today... or of all things that have ever lived on Earth.... In the case of latter supposition it is suggested that, perhaps, its correct title should be the “first universal common ancestor”. Such reasoning raises another question: what came before LUCA? Since RNA acts both as a repository of information and as a catalyst, perhaps there were evolving entities made purely from RNAs. So, what was the nature of LUCA? Some theoretical biologists think that LUCA was only one of several designs for early life, from which a single entity capable of evolving into prokaryotes arose. Others have questioned its existence altogether; that is, some scientists maintain that there may not even have been such an entity as the LUCA at all, and that it should no longer be considered as a relevant part of evolutionary theory—this view being the central tenet of the “metabolism first hypothesis” as opposed to the “genes first hypothesis”.

However a middle ground is held by those scientists who believe that the LUCA was not a single entity, but a consortium of many “LUCA-like” entities (as was proposed by Prof. Armen Mulkidjanian—see summary report). Use of the term “LUCA-like” is deliberate because although similar to the single entity, the components of such a consortium would not have been individually “complete”—sort of proto-LUCAs, as it were. In order for these entities to move up to the next level and form a complete LUCA, they would have had to exchange genes with each other and therefore exist in close proximity, which could have been encased within “semi-permeable” bubbles of clay on the sea floor and/or floating about in small pools of water, where their concentration would have been sufficiently high enough for interaction. This would have facilitated exchange of genetic material (*i.e.*, *via* HGT). On the balance of probability and based on the evidence derived from the mechanisms used in HGT (see below), I believe it is more than likely that there was a sort of “united nations of LUCAs” in operation at the time.

The explanatory evolutionary tool of LUCA does not, however, explain the presence of viruses, in particular RNA ones. There is evidence to suggest that some viruses arose independently from an RNA-world without the intervention of LUCA and are, thus, not directly connected with the emergence of either Bacteria or Archaea. In this respect some scientists working in the RNA-world hypothesis and comparative genomic studies believe that the word “common” in the acronym LUCA be replaced with “cellular”, as it is not in common to viruses. However, it should still be noted that there were extensive exchanges of MGEs between RNA viruses and the LUCA with evolving cellular genomes. 

## 5. Evolution of LUCA

Which process of evolution (Lamarckian or Darwinian) was predominant at the time of LUCA? Although the required threshold for Darwinian evolution to become a viable mechanism is uncertain, it is highly probable that Lamarckian evolution was in operation during the LUCA period. Moreover, when a LUCA gains a nucleotide(s), gene or an operon *via* HGT it is non-Darwinian evolution. Some further evidence in support of Lamarckian evolution may be gleaned from the new sciences of epigenetics and comparative genomic studies. Epigenetics is the study of changes in gene expression and its cellular phenotypes, brought about independently of the DNA sequence present within organisms, meaning that it does not involve a change in the required nucleotide sequence within the genome. Such gene expression and cellular phenotypes are shown to be heritable and this process is akin to Lamarckian evolution. Many examples of epigenetics are known but, by way of illustration, consider the following: gene expression can normally be controlled via the action of a repressor protein that binds to the “silencer” region of the DNA, thereby preventing gene expression; the same result can be achieved by DNA methylation which would not necessarily involve the relevant genomic DNA sequence. DNA methylation could be maintained throughout the cell’s life and even through subsequent cell divisions for many generations; all the while the relevant DNA sequence would remain unchanged. Comparative genomic studies determine how certain important elements (e.g., proteins, RNAs, genes and operons) can either be preserved through biological history of species, or by the acquisition of new divergent properties thereby allowing speciation to occur. Such studies involve examining both similarities and differences that arise, and become fixed due to selection pressure, in the appropriate elements (as above) from different species and thus can yield information about the function and evolutionary processes that act on genomes. In turn, we gain insight into new discoveries such as non-coding functional elements of the genome, and perhaps even the threshold levels of mechanism of evolution.

## 6. HGT Mechanisms

What are the mechanisms by which HGT occurs? Currently these include: transduction, a process whereby a viral capsule is used to transfer genetic material from one cell to another; conjugation, a process exhibited by microbes during which a plasmid or a small piece of a plasmid from one donor cell is transferred to another recipient cell (Prof. Matxalen Llosa—see summary report); transformation, which occurs when a competent cell takes up a “naked” strands of nucleic acid from the environment—such strands of nucleic acids may not necessarily have been exuded by living entities (e.g., mitochondrion genes transferred to eukaryote chromosomes), they could also be from recently dead cells, as well as from long extinct organisms (Dr. Søren Overballe-Petersen—see summary report); gene transfer agents (GTA), which are bacteriophage-like particles containing random cellular genomic segments intended for transduction to another living recipient cell (Prof. J. Thomas Beatty—see summary report); and membrane vesicle transfer (MVT), in which small membrane sacs emanating from the surface of a cell contain genetic material for transfer to another living recipient cell. Collectively, the genetic materials being transferred are termed mobile genetic elements (MGEs) which include strands of naked nucleic acids, transposons, phages, plasmids *etc.* MGEs are important in terms of gene modification, gene duplication, gene pools, acquisition of physiological properties and/or metabolic traits that are not necessarily concordant with VGT.

Studies of HGT mechanisms in evolution represent new and frontier science in which old techniques are being revised and novel techniques are being devised in order to elucidate the genetics surrounding ancient genomes—for example the discovery that a pentaribonucleotide (GUGGC) is able to carry out aminoacylation, transacylation and peptide synthesis raises the profile of RNA in relation to the major part it played in the origin of life. In other studies it has been shown that one of the major evolutionary HGTs took place when certain genes from mitochondria [3] and chloroplasts [4] were transferred to the nuclear chromosomes of their contemporary host eukaryote, resulting in the latter’s transformation. Moreover, the fact that viruses outnumber the total of all different types of living entities on Earth and taking onto account the other four modes of HGT, one can safely surmise that HGT occurs on a gigantic scale.

## 7. Conclusions

HGT is a ubiquitous process occurring in nature, via one of the discussed mechanisms or a combination of them. It is highly probably that “RNA-organisms” and/or LUCA entities were exchanging MGEs on an extensive scale, thereby bringing about the reorganisation of genetics which underpins all living organisms today.

## References

[1] Woese C.R., Fox G.E. (1977). Phylogenetic structure of the prokaryotic domain: The primary kingdoms. Proc. Natl. Acad. Sci. USA.

[2] Cavalier-Smith T. (2002). The neomuran origin of archaebactria, the negibacterial root of the universal tree and bacterial megaclassification. Int. J. Syst. Evol. Microbiol..

[B3-life-03-00518] 3.Mitochondria are thought to belong to the group of alpha-proteobacteria.

[B4-life-03-00518] 4.Chloroplasts are thought to belong to cyanobacteria.

